# Omnivores and Vegetarians Think Alike About Taste, Familiarity, and Price of Meat and Meat Analogs

**DOI:** 10.3390/nu18020264

**Published:** 2026-01-14

**Authors:** Tommaso Querini, Marco Tagliabue

**Affiliations:** Department of Behavioural Sciences, Faculty of Health Sciences, OsloMet—Oslo Metropolitan University, St. Olavs Plass, P.O. Box 4, 0130 Oslo, Norway; s319226@oslomet.no

**Keywords:** relational frame theory, IRAP, IMPACT, sustainability, vegetarianism

## Abstract

Background/Objectives: The overconsumption of animal-derived proteins represents a threat to both the environment and our health. Although there is widespread agreement that reducing meat consumption represents a more sustainable alternative, few studies have explored the implicit relations guiding these food choices. This empirical study explores meat consumption and vegetarianism through the lens of Relational Frame Theory. It is hypothesized that people who eat meat have different relational responses to images of meat and plant-based alternatives than vegetarians. Methods: We used the Implicit Attribute Classification Task (IMPACT) to measure relational responses, testing whether omnivores find plant-based proteins (1) less tasty, (2) less familiar, and (3) more expensive than vegetarians do. We registered the response latencies and calculated D-scores from 110 participants who completed an online test. Results: The study failed to find any statistically significant differences in the IMPACT measures between omnivores and vegetarians, given our specific participants and stimuli. Conclusions: Relational responding measures offer a useful approach to understanding consumer choices. However, they are highly sensitive to the task parameters and could be enhanced by further integration with other consumer behavior models when explaining meat consumption.

## 1. Introduction

Meat consumption is a common behavior throughout the world. On average, 86% of people surveyed in 39 countries around the world said they consumed meat [1]. In 2022, it was estimated that 5% of the population was vegetarian, while the percentage was 2.9% in 2019 [2]. The current patterns of production and consumption of meat are increasingly considered unsustainable because of their high environmental impact [3]. An essential change in diet is one that moves away from animal proteins and toward more environmentally friendly sources of protein. More research directed towards how to increase the rate at which consumers substitute meat with plant-based alternatives is necessary to promote this change [4].

The overconsumption of animal proteins is a major challenge, and the current animal-based protein demand is unsustainably high [5]. Thus, the familiarity of substitutes among consumers could be decisive in tackling this problem. In order to partially tackle this issue, several studies have demonstrated how low familiarity (and awareness) contributes to general consumer reticence to accept novel foods, such as cultured meat (see [6] for a review). In another study, Rosenfeld and Tomiyama [7] surveyed 579 participants and found that omnivores consider a vegetarian diet less tasty, more expensive, and less familiar.

As a form of complex human behavior, consumer behavior consists of bidirectional relations between stimuli called relational frames, and the behavior of establishing a relational frame between two stimuli is called relational responding [8]. Frames can be divided into two categories: non-arbitrary and arbitrary. Non-arbitrary frames are frames that refer to physical properties of the stimuli (e.g., size, color, quantity) and are not necessarily mediated by language [9]. Arbitrary frames are not based on physical properties and are dependent on language [10]. Similarly to any other operant behavior, relational responding is dependent on associations learned in the past. Thus, individuals show different responses to stimuli based on their arbitrary frames.

Relational responses happen over time. A relational response to a stimulus may come quite quickly and be followed by relational responses that are lengthy, distinguishing between brief and immediate relational responses (BIRRs) and extended, elaborated relational responses (EERRs) [11]. These two types of responses are both considered forms of arbitrary and applicable relational responding, but the former operates under time pressure and occurs in the first few seconds, whereas the latter occurs afterwards. As our study focuses on BIRRs, we will use this term in the remainder of the text.

### 1.1. Relational Responding Measurements of Meat Alternatives

The Implicit Relational Assessment Procedure (IRAP) [12,13] and the Implicit Attribute Classification Task (IMPACT) [14] are two computer-based psychological measuring tools that require individuals to accurately and quickly respond to the relation between two stimuli presented on screen (e.g., “cat” and “meow”) using one of two response options (e.g., “similar” and “different”) [12].

The IRAP has been used to measure attitudes towards images of meat and vegetables among vegetarians and meat-eaters [15]. The study found that both groups had “an implicit pro-vegetable bias” [15] (p. 298), although the vegetarians’ score was twice as high. The IRAP, however, measures brief and immediate relational responses (BIRRs) to only two relational frames, “positive” and “negative”. This method provides little nuance. For instance, a person may have a BIRR to a stimulus with the word “expensive” and a significantly different BIRR to the word “tasty”. The IRAP does not allow for distinguishing between the different BIRRs.

The IMPACT represents an extension of the IRAP insofar as it allows the measurement of several BIRRs. In their experiment with 260 participants, Altenburg and Spruyt [14] explored five different relational responses (healthiness, sustainability, price, taste, and ethicality) to images of meat and vegetarian proteins. They found, similarly to Trendel and Werle [16], that these relational responses were distinct. According to the authors of that study, relational responses ought to be examined separately, as they correlate with dietary choices and purchase behavior.

Parry and Szejda [17] presented the image of a product and an attribute word below it and measured reaction time. According to their results, taste and familiarity are the primary motivators for purchasing intent. Price had only a weak positive correlation with purchase intent. However, when participants were explicitly asked what encouraged or discouraged them from buying a plant-based product, they mentioned price as the second most important factor after taste.

### 1.2. Aims and Hypotheses

Our study builds on the body of evidence on measures of relational responses described in the previous section to further explore the attributes of continued meat consumption vs. uptake of plant-based alternatives that characterize sustainable consumer behavior.

Our aim is twofold: First, we contribute to the further advancement of the theory that measuring separate brief immediate relational responses may help predict behavior better than their unidimensional measurement. This may help in designing further experiments aimed at promoting more sustainable behavior. Second, we aim to enhance our understanding of the relational responses of omnivores and vegetarians towards different products. We measure six relational responses to visual stimuli representing meat and plant-based products. The subjects are divided into two categories, omnivores and vegetarians. The research question underlying this study concerns the differences in responses to these stimuli between these two categories.

Based on the literature, the three starting hypotheses are as follows: (H1) omnivores consider plant-based products less tasty compared to vegetarians; (H2) omnivores consider plant-based products less familiar compared to vegetarians; (H3) omnivores consider plant-based products more expensive compared to vegetarians.

## 2. Materials and Methods

### 2.1. Participants

Hussey et al. [18] recommend a sample larger than 107 in order for the IRAP and similar tools to have adequate power to detect a bivariate correlation. However, this type of research methodology is characterized by a very small sample size and low power: median N = 64, power = 0.34 [19]. One hundred and ten participants were enrolled online using the Prolific platform. A post hoc analysis performed with G*Power 3.1 [20] demonstrated an achieved power of 63% based on two-tailed independent-samples t-tests whose d effect size was averaged across three comparisons (taste, familiarity, and price) with sample group sizes set at n1 = 53 and n2 = 51 and an α = 0.01. This resulted in an underpowered study, assuming the common threshold for acceptable power at 80% [21]. Palan and Schitter [22] describe Prolific as a platform where it is possible to enroll a good pool of dedicated participants for sound research in the social sciences. When registering for Prolific, users list demographic information. Researchers can then use pre-screening criteria to filter and sample participants. This study used diet choice as the pre-screening criterion. In addition, after pre-screening, all participants went through validation by answering the following question: “Do you currently follow any of the following [vegetarian, vegan, omnivore] diets?” before the beginning of the experiment. Fifty-five participants were either vegetarian or vegan, while 55 participants were omnivores. As the main objective of our study was to compare meat-eaters with non-meat-eaters, we did not differentiate among other types of meat-free diets, such as pescatarian, lacto- and/or ovo-vegetarian, vegan, flexitarian, and others. All participants were based in the United States and over 18 years old. The sample was equally divided between men and women to have a balanced sample, and this division was performed directly on the recruitment platform. The gender of each participant was not registered during the experiment, nor connected to their responses; neither was their age. However, typical Prolific respondents are more likely to be female (although this was controlled for in our study), younger than the average age of the overall United States population (often in the 18–34 range), and highly educated (holding a degree) [23]. Participants were paid the fee suggested by Prolific to participate in the experiment (i.e., at the time of writing, USD 12/h).

The experiment was registered at SIKT, the Norwegian Agency for Shared Services in Education and Research, with reference number 323105. Personal data were stored according to the guidelines provided by SIKT and internal policies to OsloMet—Oslo Metropolitan University.

### 2.2. Materials and Procedure

We implemented the IMPACT software program built using PsychoPy3 software [24]. The PsychoPy3 builder allows experiments to be designed through a Graphic User Interface. The experiments were coded in Python version 3.8.10 and automatically converted to JavaScript (ES6). In order to administer the test online, the launching platform Pavlovia.org was used. Pavlovia is a platform for storing, running, sharing, and versioning online psychology experiments. Next, the experiment was uploaded to the Prolific platform. The test was administered online through a computer.

The IMPACT procedure used was almost identical to the one described in [14]. Participants first encountered a consent form detailing the purpose and approximate duration of the study, as well as information on how to withdraw from the experiment if they wished to do so. In the form, they registered their Prolific-ID, which was stored safely and could be used by participants to withdraw their consent by contacting the first author from their Prolific email (i.e., a set of letters and numbers so that it was not possible for the research team to identify the respondents).

In each trial, participants were presented with a rectangular box situated centrally on the screen on a black background. The box contained a randomized pairing of a target stimulus and an adjective or attribute in English describing it. Four target stimuli represented packaged meat products: bacon, burgers, chicken breasts, and minced meat. Four target stimuli represented packaged plant-based products: burgers, pulled “chicken”, sausage, and minced “meat”. It was not possible to reproduce the exact stimuli due to copyright and advertisement issues: these were real packaged products, marketed mostly in North America, which included known brands for both the meat and plant-based products [25].

The three pairs of attributes representing relational frames used were as follows: tasty/yucky; familiar/unfamiliar; expensive/affordable. These factors were chosen because they influence people’s dietary preferences for meat and vegetables (e.g., [17,26]). The stimuli were shown for 1500 milliseconds before disappearing and being replaced with one of three task cues (‘YES’, ‘NO’, ‘???’) beneath the box until a response was registered. These cues are used to direct participants to either of two tasks. When the cues ‘YES’ and ‘NO’ were presented (48 trials each), participants had to press the ‘I’-key and ‘E’-key, respectively. When the participant responded incorrectly, a red X was presented underneath the task cue until the participant gave the correct response. These are called non-evaluative tasks. When the cue ‘???’ was presented (48 trials), participants performed brief and immediate relational responding (BIRR). If the stimulus and the target attribute were perceived as similar, the subjects would press the ‘I’-key for ‘YES’, whereas if they were perceived as different, they would press the ‘E’-key for ‘NO’. There was no correct response for these trials. These are called evaluative tasks. A valid key press was always followed by an inter-trial interval (ITI) varying randomly between 500 and 1500 milliseconds. The subjects were first presented with six practice trials. Practice trials consisted of six stimulus–adjective pairings, followed by a task cue. All three task cues appeared at least once. The only difference between practice trials and experimental trials was that the inter-stimulus interval was always 1500 milliseconds. Following the practice trials the subjects were presented with 144 experimental trials. During these, each possible stimulus (8) x adjective (6) x task cue (3) combination was presented once. The estimated time for completing the experiment was 10 min. For each experimental trial the program recorded the stimulus, the response given, and the response latency, or the time elapsed between the presentation of the task cue and the response.

### 2.3. Data Preparation

This is a study with a correlational design. The predictor variable is the BIRR, and diet is the criterion variable. The procedure for data preparation is the same as described by Altenburg and Spruyt [14]. All analyses were conducted using R version 4.1.2 (R Core Team, 2019) in the RStudio environment version 2022.12.0 [27], Microsoft Excel (Version 2303), and IBM SPSS Statistics (Version 31.0).

According to the IMPACT procedure, evaluative tasks (i.e., those where the participant responded to a ‘???’ cue) cannot be used to measure BIRRs and were therefore excluded from analysis. Practice trials were also discarded. If participants replied too fast or too slow, the trials were excluded: the lower and upper limits for response latencies were 300 ms and 5000 ms. Similarly to Altenburg and Spruyt [14], data sets with an error rate higher than 37.5% were excluded in the current study.

Scores were computed according to the D4 IAT scoring algorithm [28]. Some participants may have been faster than others due to an overall faster reaction time. For this reason, Greenwald et al. [28] suggest calculating a standard deviation of the practice and evaluative trials combined. This yields a D-score that can be included in statistical analyses. In accordance with the scoring algorithm, the mean response latency of correct responses was used to replace error trials, but a penalty of 600 ms was added. For each participant, the D-scores were calculated for each combination of target category (meat/plant-based) and the three attribute dimensions (taste, familiarity, price) from the trials pertaining only to the relevant target category. For instance, to calculate a score for the perceived taste of meat items, the mean response latencies of the “tasty” plant-based target trials (those with the attribute “tasty” and the cue ‘YES’ or the attribute “yucky” and the cue ‘NO’) were subtracted from the mean response latencies of the “yucky” plant-based-target trials (those with the attribute “yucky” and the cue ‘YES’ or the attribute “tasty” and the cue ‘NO’) and divided by the standard deviation of latencies across all plant-based-target taste trials. Six D-scores were calculated for each participant, measuring their BIRRs to meat and plant-based stimuli for “taste”, “familiarity”, and “price”.

Positive scores indicated that the targets were perceived as tasty, familiar, or expensive. Negative scores indicated the opposite, namely, that the targets were perceived as yucky, unfamiliar, or affordable. H1 was considered supported if the D-score for taste of plant-based stimuli was significantly lower for omnivores than for vegetarians. H2 was considered supported if the D-score for familiarity of plant-based stimuli was significantly lower for omnivores than for vegetarians. H3 was considered supported if omnivores’ D-score on the dimension of price was significantly higher than that of vegetarians. Mean and standard deviation were calculated for the D-scores. Two-sample t-tests were run to test whether the differences between the diet groups were significant. The null hypothesis featured no significant difference between groups. The significance level was set at *p* ≤ 0.01, as in Altenburg and Spruyt [14].

## 3. Results

### 3.1. Attribute Testing

Based on the response validity criteria specified above, the datapoints of three participants were excluded from the omnivore group, and two participants were removed from the vegetarian group, resulting in a total of N = 104, divided into n = 53 valid measures of omnivore participants and n = 51 valid measures of vegetarian participants.

In order to compare the BIRRs to meat and plant-based stimuli, we determined whether the participants perceived a significant difference between meat and plant-based attribute categories. All three tested comparisons failed to meet statistical significance (*p* ≤ 0.01). Meat products had higher D-scores for familiarity (M_meat_ = 0.16, SD = 0.51; M_plant-based_ = 0.08, SD = 0.57, *p* = 0.257) and price (M_meat_ = 0.05, SD = 0.54; M_plant-based_ = −0.11, SD = 0.54, *p* = 0.027 (price would have been significantly different if we had set the statistical significance level at *p* ≤ 0.05)). The D-score for taste was almost identical for meat (M = 0.14, SD = 0.59) and plant-based (M = 0.15, SD = 0.62) stimuli (*p* = 0.882).

To test whether the three attributes were indeed distinct dimensions, we conducted a bivariate correlation analysis following the procedure of Altenburg and Spryut [14] among taste, familiarity, and price across both omnivores and vegetarians. The results are depicted in Table 1. Although we found positive correlations between all three dimensions, none of them were statistically significant. Moreover, the shared variance was very low: it ranged from 2.1% for taste and price to 2.3% for taste and familiarity, suggesting that the dimensions were distinct.

### 3.2. Hypothesis Testing

In this section, we report the results based on testing whether our three hypotheses were supported by the data. According to H1, omnivores consider plant-based products less tasty than vegetarians do. First, we ran a Levene’s test to check for equality of variances and found that the variances were equal between omnivores and vegetarians (F = 0.654, *p* = 0.421). Indeed, omnivores (M = 0.10, SD = 0.56) yielded lower scores than vegetarians (M = 0.21, SD = 0.67) on taste. However, this difference was not statistically significant (*t*(102) = −0.898, 99% CI [−0.434, 0.213], *p* = 0.372, *d* = 0.627).

According to H2, omnivores consider plant-based products less familiar than vegetarians. Levene’s test confirmed that equal variance between groups could be assumed (F = 0.017, *p*= 0.897). Instead, omnivores (M = 0.08, SD = 0.57) scored slightly higher than vegetarians (M = 0.07, SD = 0.58), contradicting the direction of H2. However, this difference was not statistically significant (*t*(102) = 0.129, 99% CI [−0.283, 0.312], *p* = 897, *d* = 0.577). Moreover, the D-scores for familiarity were approximately zero for both groups, suggesting that the stimulus was not considered strongly “familiar” or “unfamiliar” (the median D-score for vegetarians was 0).

Lastly, according to H3, omnivores consider plant-based products more expensive than vegetarians. The hypothesis was considered supported if the D-score for price of plant-based stimuli was higher for omnivores than for vegetarians. Equal variance between omnivores and vegetarians was assumed based on the results of Levene’s test (F = 0.005, *p* = 0.945). Both omnivores and vegetarians had a negative D-score for the price attribute, suggesting that neither of the stimulus categories was considered expensive. The score for omnivores (M = −0.18, SD = 0.53) was slightly higher than for vegetarians (M = −0.04, SD = 0.57), although this difference was not statistically significant (*t*(102) = −1.238, 99% CI [−0.412, 0.148], *p* = 0.219, *d* = 0.544). The results of the three independent-samples *t*-tests are summarized in Table 2 for improved readability and comparison.

## 4. Discussion

This study aimed at exploring BIRRs among omnivores and vegetarians by using the IMPACT as our selected experimental procedure. We measured differences in relational responding between the two groups and compared the results to other surveys and experiments. We did not find any statistically significant differences between groups for any of the three tested hypotheses (i.e., taste, familiarity, and price). Our findings contribute to further advancing the theory that measuring separate arbitrary relational responses may help predict behavior better than unidimensional measurement of brief and immediate relational responding.

The initial hypotheses were formulated based on previous studies [7,17], and given our null findings, we can interpret them in different ways. First, the subjects performing the test may have been affected by other factors. For example, the motivational state [29] (p. 61) of certain participants may have been one of hunger, and this may have affected their responses to the test. A problem with internet-based tests is that one cannot control such conditions. However, both Barnes-Holmes et al. [15] and Altenburg and Spruyt [14] had statistically significant results but had not controlled for motivational states in their studies.

Other experiments using online tests [14,17] showed significant results when comparing reaction times to visual stimuli among omnivores and vegetarians. Although the lack of statistical significance in our study may be due to a lack of sufficient power to detect any differences between omnivores and vegetarians, other potential difficulties concern the methods used in the present study. Concerning the recruitment of participants, Prolific has put in place different mechanisms to encourage high-quality submissions [22]. The web hosting platform Pavlovia is widely adopted for online experiments within the social and behavioral sciences (see [30]) and was also the platform used by Altenburg and Spruyt [14], whose basic materials and procedure were replicated in our study.

Based on our findings, vegetarians and omnivores did not have significantly different relational responses for the relational frames when presented with images of meat and plant-based proteins. Their consumption may be affected by other factors. It may be that participants had a learning history related to the specific products that were presented in the test, and this may have significantly affected the results. For example, the median D-score for familiarity of plant-based products among vegetarians was 0. An interpretation of this result is that roughly half of the participants considered these products familiar, whereas others may not have heard of them before. One of the advantages of using the IRAP methodology is that it uncovers biased or socially undesirable responses linked to meat-eating or plant-based diets in certain cultures or milieus. For example, participants in a previous IRAP study did not self-report socially punished relational responses such as attraction for children, racism, or religious and social stereotyping, indicating that BIRRs were not consistent with EERRs measured with self-reports “when participants are required to confirm verbal biases of a psychologically sensitive nature” [11] (p. 109).

Alternatively, abstinence from meat consumption could be a form of rule-governed behavior. A rule is, according to Skinner [31], a stimulus describing a contingency of behavior and its consequence. In this case, for example, “If you avoid eating meat, you are behaving ethically”. Individuals may perceive that abstaining from meat consumption may be socially reinforced by their group (i.e., a pliance; see [32] (p. 203)). Consumer acceptance is the main factor behind consumers’ choices of food products, and taste, texture, and price are the most impactful on the appeal of plant-based meat products [33]. On the other hand, the adoption of plant-based and insect-based proteins is hindered by dietary factors and “the experiential importance of meat and food neophobia” [34]. Thus, vegetarianism can be interpreted by applying the rules for individuals to eliminate or reduce the likelihood of eating meat. Self-management [29] (p. 586) would then be one strategy to implement this rule and would involve an individual’s successful use of behavioral change tactics.

Lastly, meat consumption can be viewed as a complex cultural practice [35] and, as such, is “influenced by numerous interconnected factors including individual preferences such as taste, meal timing, and social interactions, alongside external elements like affordability, cultural norms, marketing, and policy environments” (p. 1). For example, Ruby et al. explored attitude differences between vegetarians and omnivores in Western countries and in India related to animal welfare and environmental sustainability [36]. Moreover, the rising phenomenon of cultural homogenization entails a narrowed range of offered and demanded meat and meatless products (e.g., eating the same small number of staple crops). Cultural homogenization refers to the process of losing cultural variation as global and dominant practices take over local ones. Although the food offerings in virtually all individual countries have become more diverse, global diets are becoming more homogenized worldwide, which bears dangerous implications [37]. After years of growth, investments in the plant-based protein sector plummeted by 28% in 2023, and sales patterns have slowed [38]. Plant-based meat is generally still priced higher than conventional meat, and this represents a hurdle in market penetration (in addition to taste and texture) [39]. For example, Beyond Meat is a US-based alternative to meat substitutes that has gained leverage in changing consumers’ tastes and preferences, mostly through retail presence and partnerships with fast-food restaurants [40]. Seventy-one percent of US-based meat-substitute consumers interviewed were familiar with the brand [41], which reflects a generalized understanding among the American population of plant-based meat (86% according to a recent report by the Good Food Institute [42]). Our median D-scores of around zero concerning the familiarity of plant-based products may reflect the modest market for these products compared to the overall meat business, despite their growing popularity [43]. From another angle, as the United Kingdom also recently experienced a significant drop in sales of alternative protein sources, the experimental work of Naughton et al. [44] suggests emphasizing the taste and nutritional values on the front-of-pack labeling.

Consumer behavior can be approached through the study of explicit attitudes, often the focus of cognitive psychology, or implicit attitudes, whose measurement targets fast, unintentional, uncontrollable, or unconscious appraisals [45]. Implicit attitudes may play an important role in guiding purchase decisions in situations where the person is under time pressure, for example, while buying groceries at the supermarket [46]. Vermeir et al. [47] maintain that a research agenda directed towards promoting sustainable food consumption should try to understand how implicit attitudes can help predict consumer behavior.

### 4.1. Replication and Stimuli Validation

In the past few years, there has been clear evidence of publication bias and suboptimal levels of attempted study replication in the social sciences [48]. The two authors describe a results paradox, where, on the one hand, scientists are required to be objective investigators and not tamper with results and, on the other hand, are not granted publication if they do not present significant and positive findings. With the intention to counteract this second trend, this study applies an existing procedure with the use of new visual stimuli and attributes. Sidman [49] presents different forms of systematic replication. Replication by affirming the consequent starts from the statement “If A is true, B is true”. If we are able to demonstrate through an experiment that B is true, it would be a logical fallacy to affirm that A is true by default. However, Sidman argues that performing such experiments allows us to increase our knowledge about A. By performing this experiment, we have learned more about the measurement of BIRRs and whether they can give us any insight into why people make certain dietary choices.

The stimuli that were used were pictures of existing meat and plant-based products in their packages. In the original experiment, Altenburg and Spruyt [14] used pictures of actual food (i.e., tofu, quinoa, falafel, and seitan) rather than packages. Along this line, Parry and Szejda [17] showed that omnivores consider such products less tasty and are less willing to substitute meat products with these alternatives, and maintained that meat substitutes such as the ones represented in this study are a more viable alternative for omnivores. In fact, some plant-based products are similar to meat alternatives, raising difficulties among the participants in distinguishing between the two categories. Another reason why we chose packaged food was that individuals in a supermarket purchase packaged products. In addition, there is already some research [17] that used packages to measure relational responding, and in order to compare results, it was necessary for the stimuli to be similar. Packages also allow participants to identify brands that are known in the United States.

One of the assumed properties of relational responding is transformation of function—the implication that relational responding affects other behaviors, such as choosing a product (i.e., purchasing). For this reason, it was considered relevant to study relational responding in order to gain insight into what characteristics of food products influence choice. However, there are general concerns that implicit assessment measures are not good predictors of purchasing behavior. In a study using supermarket purchase data, Panzone et al. [50] found that IAT scores are not good predictors of sustainable purchases. It must nevertheless be pointed out that the IAT performed during that experiment was meant to capture participants’ relational responses related to the frame of “sustainable” and “unsustainable”. The visual stimuli presented for the relational frame “unsustainable” were images of landfills, garbage bins, bottled water, labels of products transported by air, and a picture of a meat product. The visual stimuli for the relational frame “sustainable” included three images of vegetables, an image of an energy-saving light bulb, an image of a reusable shopping bag, and an image showing a recycling box. It could be argued that the stimuli used in this study were not meant to capture relational responses that are specific to the purchase of meat products or plant-based proteins, which are the focus of this study.

### 4.2. Limitations

The first limitation of our study concerns methodological issues, including a relatively limited sample size, which is still larger than those in most other research published on relational responding, with a median of 64 respondents in IRAP research [19]. While Prolific clearly possesses several advantages as an online platform, including speed, wide reach, comparability to lab studies [51], and increased variability of respondents compared to research conducted on undergraduate students, it also comes with limitations [23]. In addition to the skewed pool of participants towards Western, Educated, Industrialized, Rich and Democratic (WEIRD) representatives, online participants are also known to be biased based on their rapid response times to newly recruiting studies, their interest in the topic, the length of the time commitment, and the pay they will receive, which may mean that maximum pay-per-hour outweighs response quality, at least among a minority of users [23]. Secondly, our study was clearly underpowered and resulted in a 37% chance of Type II errors, which may apply in our case, as we failed to reject the null hypothesis when it is indeed false. The number of participants that should be recruited, including for other forms of relational responding testing (our addition), should not only depend on the effect size estimates and α levels input into a power analysis calculation (see [19]) but also be determined based on the inference method, area, scope, and aim. For example, the author of that study reported that the sample size recommendation reported in the abstract of Vahey et al. [52] was mistakenly cited in several subsequent studies as an indication of sample size (i.e., between 29 and 37), although different analyses were performed.

Another limitation relates to the specificity of the stimuli used for meat and plant-based product alternatives and the measures of relational responding programmed in the task. Although these represent units of behavior in their own right, they are proxies of actual purchasing behavior in a consumer setting, which highlights the lack of one of the purposes of behavior analysis as a science (i.e., control) [53]. In fact, we found that images representing meat and plant-based products were not perceived as different in a statistically significant way, which could have been addressed by running pre-tests with a smaller group of participants. The lack of a statistically significant difference may invalidate the results of the study. However, it may also be the case that the participants considered images of meat and these meat-like products as similar in terms of familiarity, price, and taste.

A third limitation is that in the experiment, the participants’ genders were not recorded. Altenburg and Spruyt [14] maintain that it is well known that gender plays a role in the likelihood of consuming meat, but it is not possible to account for that in this study. This was clearly a shortcoming in the design of the web-based procedure; motivated by the fact that this study did not receive any funding for covering Prolific participation costs, we tried to keep the completion time as short as possible. Thus, we did not have the opportunity to measure any other variable in addition to response latency. For example, adding a survey to the experiment would have allowed us to gain more knowledge about the informants. We could have found out how long they had been vegetarian or how often they ate meat, what they believe are the main hindrances to becoming vegetarian, and so on. In addition, we could have measured whether there was a correlation between EERRs and BIRRs, similarly to what Barnes-Holmes et al. [15] did in their study.

“Positive”, “tasty”, and “healthy” in regard to food stimuli are three distinct relational frames with distinct BIRRs [16]. Even more significantly, according to Trendel and Werle [16], a BIRR between the stimulus and the word “positive” does not predict whether the subject picks that stimulus among others. The subject may respond that a type of food “is similar to” the word “positive”, but this does not correlate with the person choosing that food. The relational frame “tasty” was a better predictor of choice behavior. These findings indicate that a nuanced, multidimensional measuring of BIRRs is necessary to avoid false conclusions about the relationship between generic BIRRs and food choice. Different relational frames may affect people’s behavior in different ways: for example, someone may arbitrarily attach a much higher value to the affordability of a food product than to its taste. This may affect their behavior when presented with a novel product at the grocery store. As a result, conclusions drawn from looking into individual relational responses are more likely to be wrong.

Lastly, we were not able to distinguish between any possible differences between respondents who declared they followed a vegetarian diet and those who declared they followed a vegan diet, as we pooled these responses into the same group (vegetarians), as we were interested in comparing the relational responding of omnivores with that of vegetarians. However, there are several other types of diets that do not feature meat consumption and may nevertheless affect plant-based food attributes and perceptions, thus influencing consumption. For example, based on results of a 24 h cross-sectional dietary recall in Norway, Tonheim et al. found that consumption of meat and dietary substitutes among vegans, vegetarians, and pescatarians was high (ranging from 90% to 68% and 64%, respectively), with soy, oats, and peas among the prevalent raw ingredients [54].

## 5. Conclusions

Through this work, we aimed to expand our understanding of multiple attributes of meat and plant-based products and how they may affect consumer choices in an experimental procedure with high face validity. We also expected to extend these findings by hypothesizing a significant difference in responses between omnivores and vegetarians, but this was not confirmed for any of the three assessed attributes (i.e., taste, familiarity, and price.

Meat consumption is a pressing environmental issue, and the research community is in need of well-designed models, tools, and interventions to promote sustainability on a global basis. In our study, we found that the BIRRs between omnivores and vegetarians did not differ significantly among 104 respondents on the chosen set of meat and plant-based stimuli. Alternative explanations of our findings include the role of cultural homogenization, low power, and broad dietary categories.

RFT can make a meaningful contribution to this important process, but it is also one among several behavior-focused approaches to interpreting consumer choices. A new study using an updated version of the theory [55] may be useful in this regard. Other interventions based on behavior analytic principles explore how to increase the likelihood of people purchasing and consuming vegetarian products instead of meat products. For example, Martinko [56] developed an organizational behavior model of consumer behavior that could be applied to meat consumption. While the intervention proposed in his study has been replicated [57], his model has not been referred to since the publication of the article. Conversely, the Behavioral Perspective Model [58] is a more well-known approach within the field of consumer behavior analysis. As our study found some limits to the applicability of relational responding models based on response latency, we are eager to see further research on sustainable behavior that integrates the models of Martinko and Foxall. Specifically, we maintain that the role of organizations in influencing consumer behavior, and the role of cultural influences in shaping (sustainable) dietary practices, should be included in a systemic analysis of how they might influence (and be influenced by) consumers’ choices. As Xavier et al. [43] put it, “Convincing people to try new foods is extremely difficult, especially when those new foods are replacing things that they already love and that constitute a large portion of their diets, such as meat” (p. 10).

## Figures and Tables

**Table 1:** Pearson correlations of appraisals by dimension.

		D_Taste	D_Familiarity	D_Price
D_taste	Pearson Correlation	1	0.069	0.064
	Sig. (2-tailed)		0.319	0.360
	Sum of Squares and Cross-products	76.593	4.777	4.419
	Covariance	0.370	0.023	0.021
	N	208	208	208
D_familiarity	Pearson Correlation	0.069	1	0.072
	Sig. (2-tailed)	0.319		0.304
	Sum of Squares and Cross-products	4.777	61.804	4.457
	Covariance	0.023	0.299	0.022
	N	208	208	208
D_price	Pearson Correlation	0.064	0.072	1
	Sig. (2-tailed)	0.360	0.304	
	Sum of Squares and Cross-products	4.419	4.457	62.620
	Covariance	0.021	0.022	0.303
	N	208	208	208

Note. N = 208 datapoints from 104 participants (all attributes pooled).

**Table 2:** Independent-samples tests for omnivores and vegetarians.

		*t*-Test for Equality of Means							
		t	df	Significance		Mean Difference	Std. Error Difference	99% Confidence Interval of the Difference	
				One-Sided *p*	Two-Sided *p*			Lower	Upper
D_taste_veg	Equal variances assumed	−0.898	102	0.186	0.372	−0.110476	0.123080	−0.433548	0.212595
	Equal variances not assumed	−0.894	97.116	0.187	0.373	−0.110476	0.123525	−0.435028	0.214075
D_familiarity_veg	Equal variances assumed	0.129	102	0.449	0.897	0.014636	0.113208	−0.282524	0.311795
	Equal variances not assumed	0.129	101.637	0.449	0.897	0.014636	0.113254	−0.282665	0.311936
D_price_veg	Equal variances assumed	−1.238	102	0.109	0.219	−0.132065	0.106681	−0.412090	0.147960
	Equal variances not assumed	−1.237	101.638	0.109	0.219	−0.132065	0.106724	−0.412222	0.148092

Note. The variables being tested in the present table are taste, familiarity, and price of plant-based (veg) stimuli. df = degrees of freedom.

## Data Availability

The raw data supporting the conclusions of this article are openly available in OSF at https://osf.io/f3jt8/.

## Abbreviations

The following abbreviations are used in this manuscript:

BIRRs	Brief and immediate relational responses
EERRs	Extended and elaborated relational responses
IAT	Implicit association test
IMPACT	Implicit attribute classification task
IRAP	Implicit relational assessment procedure
RFT	Relational frame theory
